# Feasibility of ^15^O-water PET studies of auditory system activation during general anesthesia in children

**DOI:** 10.1186/s13550-018-0362-z

**Published:** 2018-02-05

**Authors:** Martin Mamach, Florian Wilke, Martin Durisin, Frank A. Beger, Mareike Finke, Andreas Büchner, Barbara Schultz, Arthur Schultz, Lilli Geworski, Frank M. Bengel, Thomas Lenarz, Anke Lesinski-Schiedat, Georg Berding

**Affiliations:** 1 Department of Nuclear Medicine, Hannover Medical School, Carl-Neuberg-Str. 1, 30625 Hannover, Germany; 2 Cluster of Excellence Hearing4all, Hannover Medical School, Hannover, Germany; 3 Department of Medical Physics and Radiation Protection, Hannover Medical School, Hannover, Germany; 4 Department of Otolaryngology, Hannover Medical School, Hannover, Germany; 5 Department of Anesthesiology and Intensive Care Medicine, Hospital Diakovere Annastift, Hannover, Germany; 6 Department of Anesthesiology and Intensive Care Medicine, Hannover Medical School, Hannover, Germany

**Keywords:** ^15^O-water PET, Scan duration, Smoothing filter kernel, Cut-off for statistical inferences, Cochlear implant, Promontory needle electrode, Auditory cortex activation, Anesthesia, EEG monitoring

## Abstract

**Background:**

^15^O-Water positron emission tomography (PET) enables functional imaging of the auditory system during stimulation via a promontory electrode or cochlear implant, which is not possible using functional magnetic resonance imaging (fMRI). Although PET has been introduced in this context decades ago, its feasibility when performed during general anesthesia has not yet been explored. However, due to a shift to earlier (and bilateral) auditory implantation, the need to study children during general anesthesia appeared, since they are not able to cooperate during scanning. Therefore, we evaluated retrospectively results of individual SPM (statistical parametric mapping) analysis of ^15^O-water PET in 17 children studied during general anesthesia and compared them to those in 9 adults studied while awake. Specifically, the influence of scan duration, smoothing filter kernel employed during preprocessing, and cut-off value used for statistical inferences were evaluated. Frequencies, peak heights, and extents of activations in auditory and extra-auditory brain regions (AR and eAR) were registered.

**Results:**

It was possible to demonstrate activations in auditory brain regions during general anesthesia; however, the frequency and markedness of positive findings were dependent on some of the abovementioned influence factors. Scan duration (60 vs. 90 s) had no significant influence on peak height of auditory cortex activations. To achieve a similar frequency and extent of AR activations during general anesthesia compared to waking state, a lower cut-off for statistical inferences (*p* < 0.05 or *p* < 0.01 vs. *p* < 0.001) had to be applied. However, this lower cut-off was frequently associated with unexpected, “artificial” activations in eAR. These activations in eAR could be slightly reduced by the use of a stronger smoothing filter kernel during preprocessing of the data (e.g., [30 mm]^3^).

**Conclusions:**

Our data indicate that it is feasible to detect auditory cortex activations in ^15^O-water PET during general anesthesia. Combined with the improved signal to noise ratios of modern PET scanners, this suggests reasonable prospects for further evaluation of the method for clinical use in auditory implant users. Adapted parameters for data analysis seem to be helpful to improve the proportion of signals in AR versus eAR.

## Background

Functional imaging of brain activation due to visual or auditory stimuli using radioactive biomarkers of blood flow or metabolism has been implemented for the first time in the 1980s [1–4]. With the advent of fMRI in the 1990s, most research in this context especially in healthy subjects shifted to this method avoiding radiation exposure [5, 6]. However, in adult patients with cochlear implants, radioactive biomarkers still retained their role in research since fMRI is not possible for safety reasons [7, 8]. In the last 25 years, the field of brain stimulation in general has witnessed an exponential growth in clinical applications—including diverse neurological, psychiatric, behavioral, and cognitive conditions—and research investigations [9]. Functional imaging using PET provided substantial evidence with respect to the mechanisms of action in these therapies [10]. Regarding auditory implants especially the two developments have occurred in parallel: (i) it has been recognized that implantation in children at an earlier age provides the best outcomes, since it takes advantage of sensitive periods of auditory development [11] and (ii) new approaches with respect to targets more central in the auditory pathway (brainstem, midbrain) have been developed [12, 13]. In consequence to the second development, functional imaging using PET and SPECT has been used as a monitoring tool helping to understand functional changes during auditory rehabilitation in adult users of novel types of implants [14–17]. However, small children with auditory implants have not yet been included in such studies due to their inability to cooperate in functional imaging while awake. This raises the question if those functional imaging studies are feasible during general anesthesia to circumvent this and how reliable such investigations would be.

Functional imaging studies during general anesthesia have been done in adult healthy subjects during auditory stimulation with words using fMRI [18–20]. Specifically, these studies revealed a reduced but maintained activation in auditory regions of the superior temporal cortex especially during light anesthesia while results were heterogeneous during deep anesthesia. Moreover, activations related to auditory stimuli in the auditory networks beyond the temporal cortex remained present at a light state of anesthesia [19]. Nevertheless, higher levels of auditory processing in the brain such as comprehension and memory were clearly impaired during anesthesia [18–20]. With respect to the used anesthetic, there is evidence from patients undergoing cardiac surgery that in opioid (fentanyl)-based general anesthesia (combined with the benzodiazepine flunitrazepam): (i) auditory evoked potentials are similar to the awake state and (ii) implicit memories of auditory stimuli can be registered in a higher proportion as compared to other combinations of anesthetics [21]. Correspondingly, studies in non-human primate showed with electrophysiological methods using auditory stimuli during opioid-based anesthesia activations of the primary auditory cortex and belt areas [22]. In accordance with these findings, opioid-based anesthesia has been recommended for intraoperative monitoring of cochlear implant function [23].

In parallel to increasing challenges for functional imaging with PET in patients receiving electrical stimulation therapy to their brain, a substantial improvement in imaging technology occurred, with respect to sensitivity, spatial resolution, and signal to noise ratio [24, 25].

Against this background, we systematically reanalyzed PET auditory activation studies of patients obtained in the context of patient care at Hannover Medical School. The studied patient population encompassed (awake) adult patients as well as a group of (anesthetized) children receiving functional ^15^O-water PET with auditory stimulation during general anesthesia. Besides generating for the first time data on the feasibility of this approach (PET auditory activation studies during general anesthesia), we intended to optimize the procedure.

There are several issues with respect to PET acquisition and data analysis, in which different parameters have been suggested for activation studies with ^15^O-water. These parameters are selected empirically without systematic reasoning and differ from one PET center to the other. One parameter is the acquisition duration. Early studies used relatively short durations of 40 s [2, 4]. In many studies up to now, intermediate durations between 60 and 90 s are employed [14, 17, 26–31]. Working groups from Japan often use 120 s acquisition time [8, 32–34].

A further issue is smoothing of the three-dimensional (3D) data set during preprocessing. Filter kernels between 1 and 2 times of the spatial resolution (FWHM, full width at half maximum) of the used PET scanner (7–12 mm) tended to be more frequently employed [15, 17, 27–31] as compared to kernels between 2- and 3-fold the FWHM (15–20 mm) [14, 35–37].

Finally, the cut-off used for statistical inferences from statistical parametric maps is variable. Although a *p* value less than 0.001 uncorrected for multiple comparisons is frequently employed [14, 15, 29, 38], less rigid thresholds have been applied as well [28, 30, 31, 37].

Consequently, the aims of the present study are to (i) explore the feasibility of functional imaging studies of the auditory system with PET in children during general anesthesia, (ii) assess the impact of the acquisition duration after ^15^O-water application on the statistical outcome measures, and (iii) elaborate adapted parameters for ^15^O-water PET studies of auditory cortex activation with respect to pre-filtering and cut-off for statistical inferences — in particular for impaired activations during general anesthesia.

## Methods

### Patients

Altogether, 26 patients with severe bilateral inner ear hearing loss were enrolled in this study. The entire group splits into one subgroup of children (10 male, 7 female) with a mean age of 6.3 ± 4.7 years and one subgroup of adults (4 male, 5 female) with a mean age of 45.0 ± 26.1 years. In the children’s subgroup, 13 received unilateral cochlear implantation 4.5 ± 2.4 years before and four 0.5 ± 0.4 years after ^15^O-water PET. Twelve children received a second implant 0.6 ± 0.4 years after the PET investigation. In the adult’s subgroup, all received their first implant after the PET study (0.4 ± 0.2 years) and 3 received a second implant 2.4 ± 1.0 years after the PET study. Cochlear implants (CI) from different manufacturers were implanted (Cochlear Nucleus®, Advanced Bionics Clarion™, and HiRes90K™, MED-EL Pulsar and Sonata). Final follow-up assessments of hearing capabilities were obtained in children 6.1 ± 0.8 years and adults 5.6 ± 2.3 years after the PET investigation. The speech intelligibility was scored between 0 and 100% for each of four tasks: (i) understanding of monosyllables at 65 dB, (ii) numbers at 65 dB, (iii) the Hochmair-Schulz-Moser (HSM) sentence test during silence, and (iv) the HSM sentence test with 10 dB background noise [39]. The mean scores achieved in children and adults are given in Table 1. No significant differences were observed between the two subgroups.

**Table 1:** Comparison of speech intelligibility 6 years after PET, between patients studied with and without anesthesia. No significant differences were observed

Age group	Anesthesia during PET	Percent understanding			
		Of mono-syllables	Of numbers	HSM sentence test	
				In silence	In 10 dB
Adults	No	54 ± 36	80 ± 33	70 ± 32	43 ± 38
Children	Yes	75 ± 11	98 ± 4	82 ± 20	37 ± 30

*HSM* Hochmair-Schulz-Moser, *dB* decibel

### Anesthesia

Auditory stimulation and PET imaging were always performed during waking state in adults and flat electroencephalography (EEG) conducted general anesthesia in children. Anesthesia was induced with sevoflurane inhalation via mask. After establishing an intravenous access, endotracheal intubation was performed under additional analgesia with remifentanil (1 μg/kg/30 s) and muscle paralysis with mivacurium. Sevoflurane was discontinued after intubation, and ventilation was continued with an air-oxygen mixture. During maintenance of anesthesia, remifentanil was given at a dose of 0.35 μg/kg/min and the sedative midazolam at a dose adjusted to achieve flat stages of anesthesia. The stage of anesthesia was assessed using the Narcotrend® monitor [40]. Based on a frontal EEG lead, this monitor allows to differentiate six stages from A = awake to F = very deep anesthesia. We targeted for stage B corresponding to an EEG pattern with dominating ß- and θ-waves in the present study.

### Auditory stimuli

Stimulations of the auditory system were always applied unilaterally: via needle electrode placed at the promontory or CI. For promontory stimulation (always applied to not implanted ears), a dedicated device (Cochlear, Germany) was set to burst stimulation with a frequency of 100 Hz and current strength between 5 and 200 mA. In adults, the current strength was adjusted according to the patient’s comfortable hearing perception; in children (i.e., during general anesthesia), a standard value of 200 mA was used. For stimulations via cochlear implant (only done in children), music was directly fed from a compact disk (CD) player to the speech processor with loudness set to maximum. All stimuli were started 30 s before injection of the radiotracer. Each auditory condition was repeated six times with 10-min intervals between tracer injections to allow for decay of radioactivity. Consequently, full PET activation studies for both ears including scans during silence as a reference require 18 scans, i.e., altogether more than 3 h for the patient lying on the examination table. For the comfort of the patients studied while awake, we divided acquisitions into two sessions of nine scans with a pause of at least 2 h in between. The conditions (A) silence and stimulation via promontory needle electrode (PN) or CI on the left (B) or right side (C) were arranged as follows: in adult patients studied while awake, A-B-B-A-B-B-A-B-B, pause, A-C-C-A-C-C-A-C-C; in children studied during anesthesia, A-B-C-A-B-C-A-B-C-A-B-C-A-B-C-A-B-C.

### Radiopharmaceutical

^15^O-Water was produced employing a Scanditronix MC35 cyclotron and administered using an advanced system as described previously [41]. For each emission scan, an age-dependent activity amount of ^15^O-water was administered always as a bolus within 7 s: up to the age of 5 years 185 MBq, up to 10 years 370 MBq, up to 15 years 555 MBq, and in adults 740 MBq.

### Data acquisition and reconstruction

For acquisition, an ECAT EXACT 922/47 (Siemens, Erlangen, Germany) PET scanner with a spatial resolution of 7 mm (FWHM) was used. Before each session of 9 or 18 emission scans, a 10-min transmission scan was acquired using ^68^Ge rod sources. Emission scanning was always started 15 s after radiotracer injection to account for circulation time and allow the tracer to arrive in the brain. Two consecutive frames of 60 and 30 s were acquired. The data were reconstructed iteratively using an ordered subset expectation maximization (OSEM) algorithm with 6 iterations and 16 subsets. The dimensions of the reconstructed 3D data sets were 128/128/47 (*x*/*y*/*z*) with a voxel size of 1.87 mm/1.87 mm/3.38 mm. Data sets of ^15^O-water uptake integrated for 60 and 90 s were used in the further analyses.

### Individual PET data analysis

Data were analyzed using the statistical parametric mapping (SPM) software (SPM8, Wellcome Trust Centre for Neuroimaging, London, UK). Data of each patient was analyzed individually (single-subject analysis). At first, all scans were realigned to one mean image, which had been obtained across all conditions, to correct for movement artifacts. Thereafter, all scans were spatially normalized using the parameters obtained with the mean image and the ^15^O-water PET template provided in SPM8. Default settings of SPM8 software were applied. As a last step of preprocessing, smoothing was applied to all scans using three different filter kernels: [10 mm]^3^, [20 mm]^3^, and [30 mm]^3^. Additionally, unsmoothed data sets (filter kernel [0 mm]^3^) were considered in further analysis. A paired *t* test was employed in all single-subject analyses to compare stimulated and reference conditions. Pairs were always built: between scan during stimulation and the closest preceding scan during silence. Three levels of significance (*p* < 0.001, *p* < 0.01, and *p* < 0.05) were applied with respect to statistical inferences. A volume of interest (VOI) template conforming the anatomical standard space and reflecting Brodmann areas (BA) was used to identify significant regional effects in primary and secondary auditory regions (AR): BA 41, BA 42, BA 22, and BA 21. The impact of data acquisition and reconstruction parameters on the demarcation of auditory activations was assessed using the following criteria: (i) the peak height (*T*_max_ value) of activation in AR, (ii) the relative size of activations within AR and within extra-auditory regions (eAR) — both in relation to the total size of AR, and (iii) the frequency of any activation in AR and eAR at all. For all assessments, left and right sides of the cortex were always combined.

### Statistical analysis across subjects

The impact of anesthesia and the abovementioned parameters selected for PET data acquisition and analysis on the demarcation of auditory activations has been evaluated based on 50 single-subject analyses (in 2 of 26 patients, only unilateral auditory stimulation had been performed). Three groups were formed for further statistical comparisons: (i) *n* = 17 studies of PN stimulation in adults during awake state, (ii) *n* = 20 studies of PN stimulation in children during anesthesia, and (iii) *n* = 13 studies of CI stimulation in children during anesthesia. Significances of differences in peak heights, sizes, or frequencies of activations between different groups or different parameters were assessed using JMP 10 software (SAS Institute Inc.) with a threshold of *p* < 0.05.

## Results

### Impact of scan duration and anesthesia on peak height in auditory cortex activations

In Table 2, mean *T*_max_ values obtained in SPM analyses based on different durations of the PET scan are compared for different conditions separately (i.e., studies with or without anesthesia and studies with auditory stimulation via PN or CI). No significant difference between the two scan durations was observed (Table 2). However, it could be observed that studies without anesthesia (in adults) showed consistently higher *T*_max_ values compared to those including anesthesia for all evaluated scan durations and smoothing kernels - *p* values indicating the significance of the respective difference are given in Table 2. Moreover, we compared *T*_max_ values between sides contralateral vs. ipsilateral to the auditory stimulation. However, no significant side differences (*p* > 0.05, paired *t* test) were found for all groups listed in Table 2.

**Table 2:** Comparison of peak height (t_max_ values) of auditory activations obtained with different scan durations (60 vs. 90s) and depending on anesthesia (no vs. yes). Mean values and standard deviations of t_max_ are given for different subgroups and smoothing kernels

Age group	Condition		T_max_ values and significance p, of difference											
			S = [0 mm]^3^			S = [10 mm]^3^			S = [20 mm]^3^			S = [30 mm]^3^		
	Anesthesia	Stimulation	60s.	90s	p	60s.	90s	p	60s.	90s	p	60s.	90s	p
Adults	No	PN	10.82 ± 2.39	12.06 ± 3.78	NS	11.68 ± 3.25	10.98 ± 5.24	NS	9.29 ± 3.91	8.54 ± 3.30	NS	7.14 ± 3.74	7.31 ± 6.57	NS
			NS	*p* = 0.0313		*p* = 0.0001	*p* = 0.0071		*p* = 0.0004	*p* = 0.0194		*p* = 0.0054	NS	
Children	Yes	PN	9.56 ± 3.59	9.37 ± 3.43	NS	6.27 ± 2.52	6.86 ± 2.49	NS	4.81 ± 2.71	5.49 ± 4.27	NS	3.90 ± 2.54	4.09 ± 2.95	NS
Children	Yes	CI	10.06 ± 3.27	9.33 ± 2.99	NS	6.80 ± 2.10	8.07 ± 3.04	NS	5.28 ± 2.24	5.47 ± 2.96	NS	3.38 ± 1.16	4.40 ± 1.97	NS

*PN* promontory needle electrode, *CI* cochlear implant, *S* smoothing kernel, *NS* not significant

### Relative sizes of activations in AR and eAR with different parameters

In Table 3, mean relative sizes of activations in AR and eAR are given for SPM analyses based on different smoothing filter kernels applied in preprocessing and cut-off levels used for statistical inferences. Moreover, mean ratios of AR to eAR size are given. Mean sizes and ratios are listed separately for different conditions. In studies without anesthesia (in adults), stimulation via PN and employing a cut-off level of *p* < 0.001 in the mean about 1% of the AR (i.e., of BAs 41, 42, 22, and 21) showed a supra-threshold activation. At the same time, activations in eAR were considerably larger (4–8% in the mean). The highest ratio between AR and eAR of 0.16 was observed at that cut-off level and with a smoothing kernel of [20 mm]^3^.

**Table 3:** Mean relative sizes of activations in AR and eAR—both given as percent of the overall size of the auditory region. Moreover, a ratio of auditory to extra-auditory activations (AR/eAR) is given (bold). The three parameters are listed for different smoothing filter kernels (*S*) and thresholds for statistical inferences (*p*). About 1% activation in AR is reached with *p* < 0.001 for studies without anesthesia and *p* < 0.01 for those with anesthesia (italic type)

				*S* = [0 mm]^3^			*S* = [10 mm]^3^			*S* = [20 mm]^3^			*S* = [30 mm]^3^		
Age group	*p* <	Anesthesia	Stimulation	AR (%)	eAR (%)	AR/eAR	AR (%)	eAR (%)	AR/eAR	AR (%)	eAR (%)	AR/eAR	AR (%)	eAR (%)	AR/eAR
Adults	0.001	No	PN	0.3	4.3	**0.07**	0.7	5.8	**0.12**	*1.0*	6.1	**0.16**	0.8	7.5	**0.12**
	0.01	No	PN	2.8	39.4	**0.07**	5.9	62.5	**0.09**	9.9	83.6	**0.12**	9.2	92.8	**0.10**
	0.05	No	PN	10.0	163.0	**0.06**	16.8	242.1	**0.07**	21.8	324.6	**0.07**	21.8	358.7	**0.06**
Children	0.001	Yes	PN	0.2	2.4	**0.05**	0.2	2.5	**0.02**	0.0	4.3	**0.03**	0.4	5.0	**0.02**
	0.01	Yes	PN	*1.1*	22.0	**0.04**	*1.2*	23.0	**0.03**	*1.3*	25.4	**0.02**	*1.6*	31.0	**0.05**
	0.05	Yes	PN	4.5	108.6	**0.04**	4.9	112.0	**0.04**	5.0	114.1	**0.03**	4.9	120.2	**0.03**
Children	0.001	Yes	CI	0.1	2.9	**0.04**	0.0	2.2	**0.03**	0.1	1.6	**0.22**	0.1	0.3	**0.15**
	0.01	Yes	CI	*1.0*	19.9	**0.05**	*1.1*	20.9	**0.07**	*0.8*	19.9	**0.07**	*0.7*	16.4	**0.05**
	0.05	Yes	CI	4.9	101.2	**0.05**	5.3	103.2	**0.06**	4.0	103.4	**0.06**	2.5	100.5	**0.05**

*AR* auditory regions, *eAR* extra-auditory regions, *PN* promontory needle electrode, *CI* cochlear implant, *S* smoothing filter kernel

**Fig. 1:**
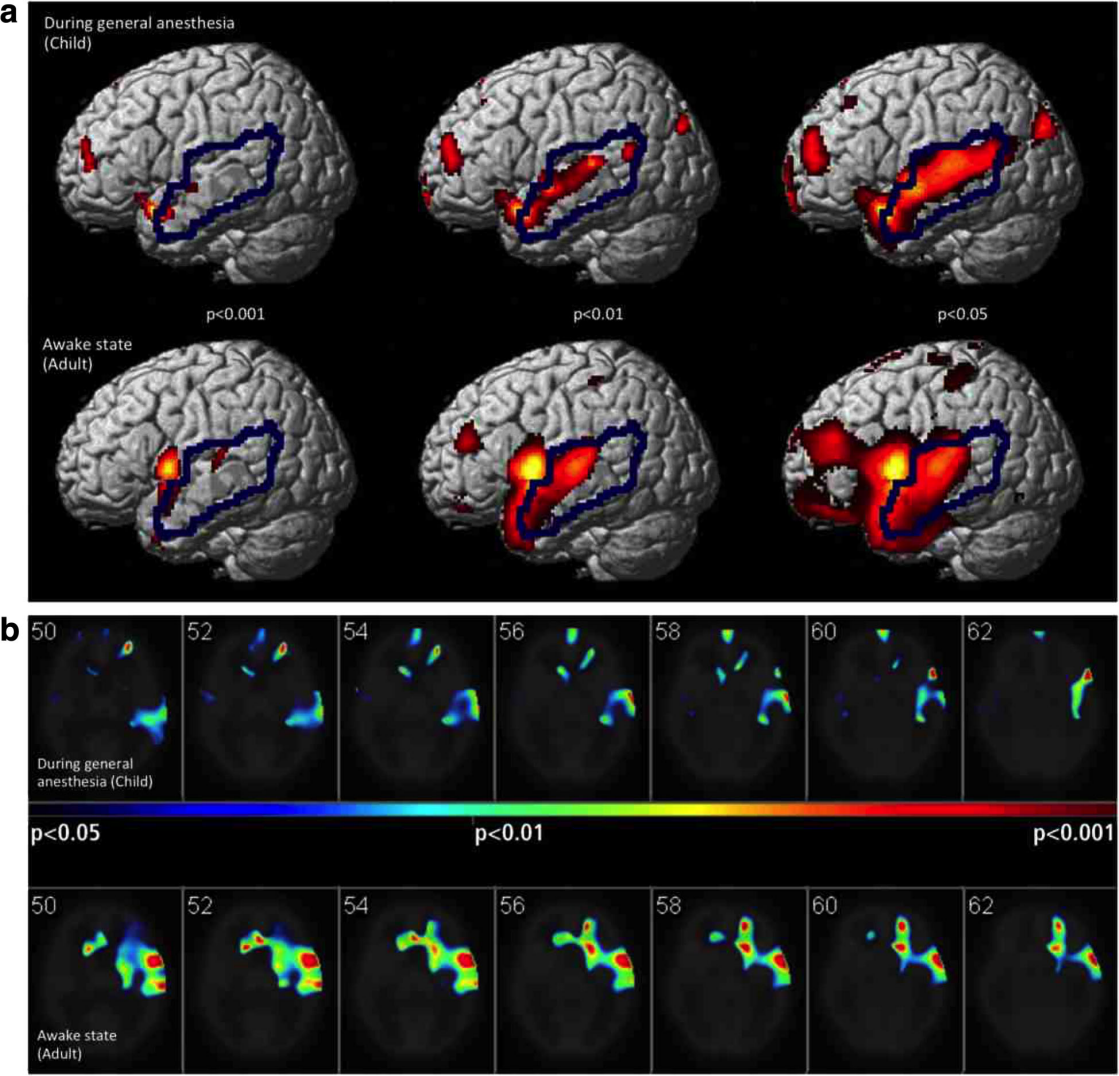
**a** Examples of activations in auditory and extra-auditory regions (AR and eAR separated by blue line), during general anesthesia in a child and awake state in an adult patient detected with different cut-off levels used for statistical inferences (all smoothed with a filter kernel of [20 mm]^3^). Stimulation was done via cochlear implant (upper row) and promontory needle electrode (lower row), respectively. Note: A realistic size of activations in AR during narcosis was only achieved using the cut-off levels of *p* < 0.01 or *p* < 0.05. At the same time, relatively less activation in eAR is seen during anesthesia compared to waking state with these cut-off levels. **b** Corresponding t-maps to the examples displayed below **a**. The t-map from the study acquired during general anesthesia in a child nicely depicts the activated auditory cortices with *p* < 0.01 (color scale green/blue), while the t-map reflecting the waking state study in an adult patient did the same with *p* < 0.001 (color scale dark red/brown). Using *p* < 0.01 for the latter would result into pseudo-activation up to the ventral frontal cortex

For studies during anesthesia (in children), the comparable mean sizes of activation in AR (around 1%) were observed with the less rigid cut-off level of *p* < 0.01. At that cut-off level, mean activations in eAR were considerably smaller during anesthesia (22–31%) compared to studies during waking state in adults (39–93%). This is illustrated in Fig. 1a, showing results of one PET study during anesthesia (upper row) and one during waking state (lower row). A realistic size of auditory activation during anesthesia was only achieved with less rigid cut-off levels (*p* < 0.01, *p* < 0.05). Moreover, at those cut-off levels, extra-auditory activations were clearly less extended during anesthesia compared to waking state. The adequacy of the respective *p* thresholds is further supported by the t-maps in Fig. 1b.

### Frequency of activations in AR and eAR depending on parameters and use of anesthesia

**Fig. 2:**
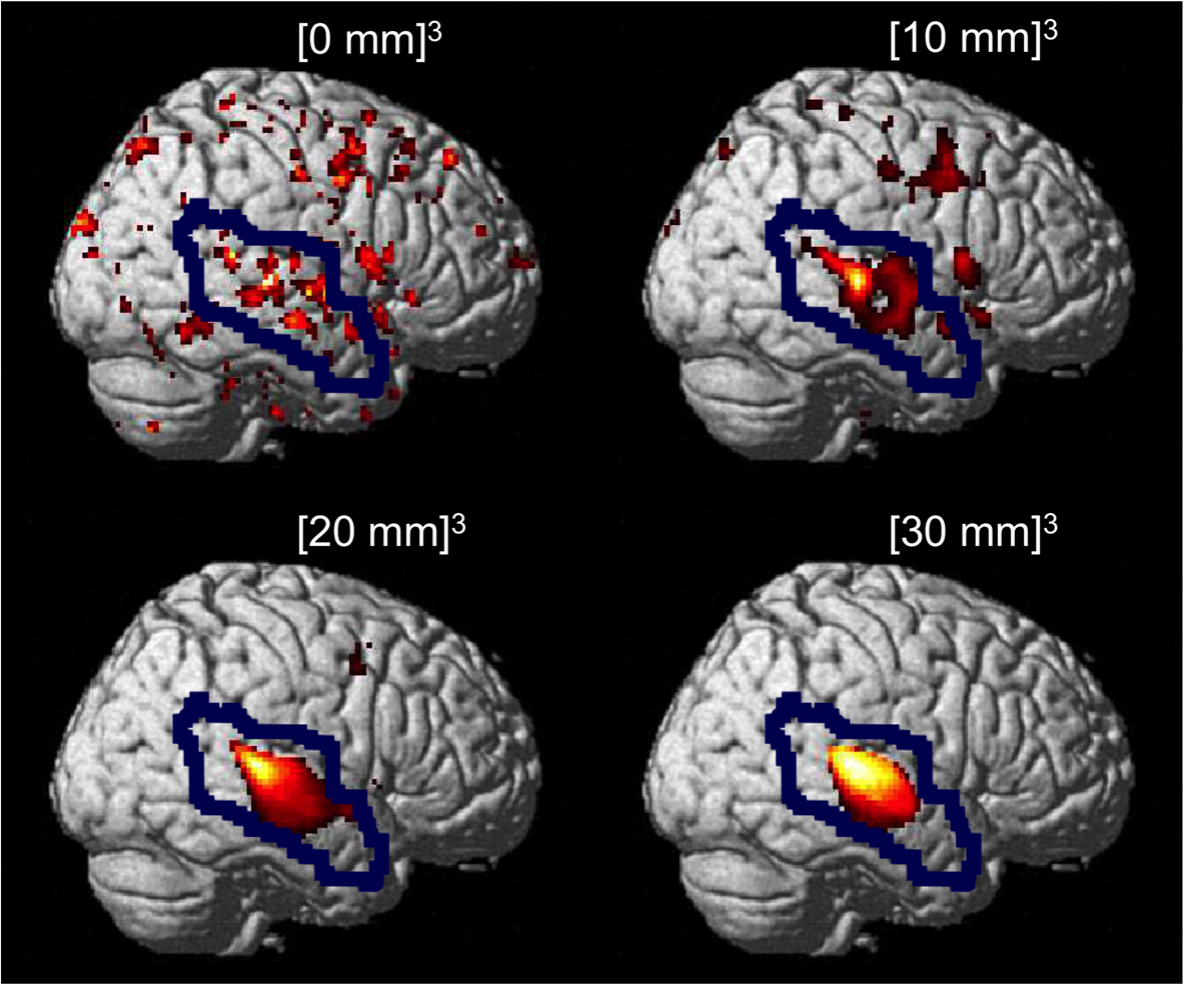
Examples of activations in auditory and extra-auditory regions (AR and eAR separated by blue line) detected with different smoothing filter kernels (always using a cut-off for statistical inferences of *p* < 0.01). Stimulation was done in an adult patient via promontory needle electrode during awake state. Note: The highest proportion of activation in the auditory cortex was detected with the second highest degree of smoothing (filter kernel of approximately three times of the full width at half maximum, [20 mm]^3^). Higher degrees of smoothing result in the detection of less extra-auditory activation

In Table 4, mean frequencies of activations in AR and eAR are given for SPM analyses based on different smoothing filter kernels applied during preprocessing and cut-off levels used for statistical inferences. Many times, for corresponding smoothing filter kernels and cut-off levels, the frequencies of activations in AR were significantly lower with anesthesia in children compared to without anesthesia in adults (e.g., with [20 mm]^3^ smoothing filter kernel and cut-off level of *p* < 0.001: 35 vs. 82%, *p* < 0.05). A more comparable frequency of activations in AR for studies during anesthesia was observed with a less ridged cut-off level (e.g., for [20 mm]^3^ smoothing filter kernel with a cut-off level of *p* < 0.01 or *p* < 0.05: 65 or 85%). For all conditions and at all cut-off levels, the frequency of detected activations in AR increased with weaker smoothing. However, activations in eAR were detected for smoothing filter kernels up to [20 mm]^3^ in 100% of the cases (for all conditions and cut-off levels). Only with the highest smoothing filter kernel [30 mm]^3^and a cut-off level of *p* < 0.001 activations in eAR were seen in less than 100% (but not less than 75% of the cases). When looking at representative SPMs obtained in one patient based on different smoothing filter kernels (always with the cut-off level of *p* < 0.01) as shown in Fig. 2, it is obvious that activation in eAR gets clearly reduced with higher smoothing filter kernels. However, the size of activation in AR increases with stronger smoothing — which can also be extracted from Table 3. Nevertheless, the difference between the highest smoothing levels is small or even reversed as can be seen in the example case (Fig. 2).

**Table 4:**
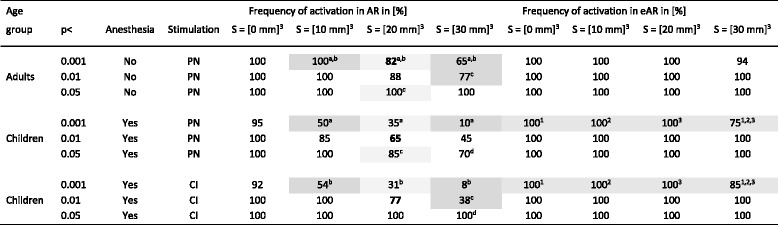
Frequency of activations in AR and eAR – dependency on selected threshold for statistical inferences and applied smoothing filter-kernel. Frequency of activation in AR around 65–82% were reached with *p* < 0.001 for studies without anesthesia and *p* < 0.01 for those with anesthesia (bold, S = [20 mm]^3^). Gray background indicates which values have been compared with respect to significance of difference (explicitly assigned by superscripts)

*AR* auditory regions, *eAR* extra-auditory regions, *PN* promontory needle electrode, *CI* cochlear implant, *S* smoothing filter-kernel

^a, b, c, d^ Significant differences within columns (t-test: *p* < 0.05), ^1, 2, 3^ Significant differences within rows (t-test: *p* < 0.05)

## Discussion

The functional performance after cochlear implantation can vary between simple sound detection and very good speech intelligibility [42]. The underlying mechanisms in the individual case often remain unclear. Furthermore, in the situation of unilateral implantation, the potential benefit of a second implant can be difficult to predict [11]. Therefore, objective functional measures reflecting individually either intactness of the central auditory pathway or correlates of central auditory processing are desirable. ^15^O-Water PET has been employed for that purpose using stimuli presented either via auditory implants or promontory needle electrodes [42, 43]. The necessity of an objective measure is given all the more in patients not able to cooperate in testing procedures of auditory function like small children.

Therefore, we investigated the feasibility of ^15^O-water PET activation studies of the auditory system during general anesthesia. During a flat opioid-based EEG conducted general anesthesia, we could demonstrate in children activations of the auditory cortex during stimulation via a promontory needle electrode or a cochlear implant. However, when applying the same parameters for PET data analysis as in awake adults, activations during anesthesia were less frequent and significant (with respect to peak height and extent). This observation corresponds to findings in healthy subjects using fMRI [18–20]. Nevertheless, it would be desirable that diagnostic accuracy of auditory activation studies during anesthesia at least partially approaches that of studies in waking state.

The present data indicate that specifically adapted parameters for PET images are helpful in this regard. Particularly, similar sensitivity can only be obtained if the cut-off for statistical inferences is lowered for studies during anesthesia. Typically, for single-subject analyses of PET studies in awake patients, an uncorrected *p* value less than 0.001 is used for cut-off [14, 44]. Other investigators have suggested less rigid cut-off values of *p* < 0.05 or *p* < 0.01 particularly for studies of the auditory system [37, 38]. One argument in favor of this is that the open hypothesis with respect to the location of detected activations typically associated with a level of *p* < 0.001 is not fitting to the situation of an auditory activation study with a high likelihood of an activation predominantly in the auditory system. We observed a similar sensitivity in the detection of auditory cortex activation during anesthesia compared to waking state using a cut-off level of *p* < 0.05 or *p* < 0.01 instead of *p* < 0.001.

Furthermore, the present study indicates that in activation studies during anesthesia, a relatively high degree of smoothing (e.g., filter kernel equivalent to 2- to 3-fold the FWHM) results into considerably less frequent “activations” in extra-auditory regions (Table 4, Fig. 2). These extra-auditory “activations” during anesthesia and stimulation via promontory needle electrode or CI (music) are unexpected and likely to be primarily artificial [18, 19, 45]. On the other side, adequate filtering to detect the expected signal in the auditory cortex (despite narcosis) might be achieved by setting it according to the matched filter theorem. This theorem suggests that the optimal smoothing filter kernel should match the spatial size of the signal to be detected [46, 47]. In auditory activation studies, the main structure in which a signal should be detected is obviously the primary auditory cortex and it should be possible to separate it from the secondary and association areas. The extent of the primary auditory cortex (Heschl’s gyrus, Brodmann area 41) might be assumed to be about 20 to 30 mm — despite a considerable inter-individual variation [48, 49]. Thus, matching the filter kernel to the extent of the primary auditory cortex according to the filter theorem results again into a relatively high degree of smoothing (equivalent to 2- to 3-fold the FWHM of the scanner used in our study). Employing this in the present study produced similar frequencies of auditory cortex activations with and without anesthesia (with the respective cut-offs). Higher frequencies of auditory cortex activations during aesthesia achieved with less smoothing filter kernels are likely to reflect in part false positive findings. This is further illustrated in Fig. 2 [0 mm]^3^ displaying activations in auditory and non-auditory regions appearing as comparable “noise” consisting of small accidentally “significant” clusters. Further evidence that the detection of a diagnostically relevant signal (in that case of reduction) is not hampered by stronger filtering comes for studies in neurodegenerative diseases. In these, it has been demonstrated for a large range of filter kernels (between 8 and 18 mm), that a higher degree smoothing had only a very small impact on the detection of the relevant signal, i.e., diagnosis [47]. Nevertheless, it has to be kept in mind that with very large filter kernels (e.g., [30 mm]^3^, also evaluated in the present study), signals might be compromised. This is illustrated by consistently lower mean *t*_max_ values observed with [30 mm]^3^ compared to [20 mm]^3^ for both acquisition durations in all subgroups (see Table 2). Moreover, too large filter kernels might be detrimental with modern generations of PET scanners — providing in particular at least twice as much spatial resolution compared to the PET scanner used in the present study. These modern scanners enable sufficient recovery of signal for substructures of the auditory system of half of the size compared to the scanner used in the present study, which could be lost by excessive smoothing.

As mentioned above, another issue potentially influencing the accuracy of ^15^O-water PET activation studies of the auditory system is scanning duration. Shorter as well as longer acquisition durations have been proposed. Kanno et al. [50] demonstrated an increase of signal to noise (S/N) ratio with acquisition time. In the mean, they found S/N ratio to be about 1/3 higher at 120 s compared to 40 s scan time. On the other hand, Volkow et al. [51] showed the largest activations in the occipital cortex due to light stimulation based on acquisitions during “uptake phase” (the initial 30–35 s). Moreover, they demonstrated that activations depend on the duration of stimulation. Continued stimulation after the peak of uptake phase for further 40 s during the “washout phase” resulted into lower uptake compared to stimulation restricted to the uptake phase. The authors explained this observation and also lower activation with longer acquisitions by increased clearance of radioactivity from areas of high blood flow. In our study, we did not observe a significant difference in activation height (*T*_max_) dependent on the acquisition duration (60 vs. 90 s). This is particularly in contrast to a study by Silbersweig et al. [52] reporting improved significance (greater signal to noise ratio and higher *Z*-score) of results obtained with 90 s compared to 60 s. One reason for these conflicting results might be differences in the injection procedure—particularly with respect to the duration of the bolus injection. Silbersweig et al. [52] described a slow bolus injection (over 20 s), which is considerably slower compared to Volkow’s and our study (3 and 7 s, respectively). A slower injection might promote improved results with longer acquisition durations. Thus, a final conclusion with respect to the most favorable scan duration is not yet possible based on the presently available data. Nevertheless, future studies employing modern PET equipment with the capability of list mode acquisition enabling flexible retrospective definition of reconstructed time frames would allow to address this issue more precisely. Furthermore, due to achievements in PET technique like time of flight measurements, an enhanced signal to noise ratio for a given acquisition duration can be expected [24]. Finally, this will help to adapt the acquisition duration rather on biological/physiological grounds than on technical limitations of the scanner for improved results of PET activation studies.

Some limitations of our study have to be considered. First, that it is a retrospective study. A prospective study would have offered the possibility, e.g., to select age-matched patients studied with and without anesthesia. Moreover, auditory stimulations were performed routinely on the left side first (without randomization); therefore, an order effect cannot be excluded. A further limitation is caused by the use of the standard SPM template for adults for spatial normalization in children. Muzik et al. [53] explored this issue. They found acceptable errors in children down to 6 years. In younger children, mean deviations of the brain contour around 2–3 mm have to be expected. However, this might be more relevant in the localization of epileptogenic foci (as in Muzik’s [53] patient population) as compared to more extended auditory cortices. Furthermore, some beneficial effect might be expected due to higher degrees of smoothing as applied in the present study. And, finally, at least visual inspection of the normalization result in all cases of the present study did not reveal any artifacts. Furthermore, with respect to the impact of the acquisition duration, it would have been desirable to compare more than two time periods. However, this was unfortunately not possible retrospectively since our scanner did not allow performing list mode acquisitions, and therefore, we had to stick with the originally used frame sequence. At last, an impact of the use of CI’s from different vendors could not be completely excluded. However, due to the facts that music was used as a robust stimulus not that dependent on the speech processor and most of the implants reflected the technique available during a relatively short period between 2001 and 2007, a major impact is not expected. Despite the abovementioned limitations, we considered it as an added benefit to obtain insights regarding the feasibility of auditory activation studies with ^15^O-water PET during anesthesia based on available data obtained on clinical grounds.

## Conclusions

The present data indicate that auditory activation studies with ^15^O-water PET in children during general anesthesia are feasible. They can approach results of studies obtained during waking state in adults if an adapted less rigid cut-off for statistical inferences (*p* uncorrected < 0.01 or *p* < 0.05) is employed and strong smoothing during preprocessing (filter kernel ≥ [20 mm]^3^) — like in studies without anesthesia. Nevertheless, further research is needed to ascertain whether activation studies with ^15^O-water PET in children during general anesthesia are a valuable diagnostic tool before and after auditory implantation in clinical routine situations. The issue of optimal scan duration is still not clarified on the basis of the presently available data. However, modern PET scanners providing fundamentally higher signal to noise ratios — in comparison to the generation of PET scanners employed to elaborated procedures for auditory activation studies with PET decades ago — will open novel possibilities to explore the circuitry of the auditory system in implant users.

## Abbreviations

^15^O: Oxygen-15
3D: Three-dimensional
AR: Auditory region
BA: Brodmann area
CI: Cochlear implant
eAR: Extra-auditory region
EEG: Electroencephalography
fMRI: Functional magnetic resonance tomography
FWHM: Full width at half maximum
HSM: Hochmair-Schulz-Moser
PET: Positron emission tomography
PN: Promontory needle electrode
SPM: Statistical parametric mapping
VOI: Volume of interest
